# Variation in the Distribution of Four Cacti Species Due to Climate Change in Chihuahua, Mexico

**DOI:** 10.3390/ijerph110100390

**Published:** 2013-12-24

**Authors:** Leonor Cortes, Irma Domínguez, Toutcha Lebgue, Oscar Viramontes, Alicia Melgoza, Carmelo Pinedo, Javier Camarillo

**Affiliations:** 1 Facultad de Zootecnia y Ecología, Universidad Autónoma de Chihuahua, Periférico R. Almada Km 1, Chihuahua, Chih. 31000, Mexico; E-Mails: evelina_p7@hotmail.com (I.D.); tlebgue@uach.mx (T.L.); amelgoza@uach.mx (A.M.); cpinedo@uach.mx (C.P.); jcamarillo@uach.mx (J.C.); 2 Facultad de Contaduría y Administración, Universidad Autónoma de Chihuahua, Circuito Universitario #1, Nuevo Campus Universitario, Chihuahua, Chih. 31000, Mexico; E-Mail: oviramon@uach.mx

**Keywords:** MaxEnt, modelation, cacti, climatic change, *Coryphantha macromeris*, *Mammillaria lasiacantha*, *Echinocereus dasyacanthus*, *Ferocactus wislizenii*, distribution

## Abstract

This study is about four cacti species in the state of Chihuahua, (*Coryphantha macromeris*, *Mammillaria lasiacantha*, *Echinocereus dasyacanthus* and *Ferocactus wislizenii*). Geographic distribution was inferred with MaxEnt. Projection was estimated under three scenarios simulated from IPCC (A2, B1 and A1B) and four periods (2000, 2020, 2050 and 2080) with 19 climatic variables. MaxEnt projects a species decrease in 2020 under scenario A2, increasing in the following years. In 2080 all species, except *E. dasyacanthus,* will occupy a larger area than their current one. Scenario B1 projected for 2050 a decrease for all species, and in 2080 all species except *E. dasyacanthus* will increase their area. With A1B, *C. macromeris* decreases 27% from 2020 to 2050. *E. dasyacanthus* increases from 2020 to 2050 and decreases 73% from 2020 to 2080. *M. lasiacantha* decreases 13% from 2020 to 2080 and *F. wislizenii* will increase 13% from 2020 to 2080. Some species will remain stable on their areas despite climate changes, and other species may be affected under the conditions of the A1B scenario. It is important to continue with studies which give a broader perspective about the consequences of climate change, thus enabling decision-making about resource management.

## 1. Introduction

In the last decades, planet Earth has been warming up and it is clear that human activities have affected the weather and the balance of the planet’s natural cycles. [1] Weather phenomena depend on a number of factors that interact in complex ways, in contrast to the traditional notion, which is the set of variables that impact directly the way the atmosphere restructures itself with a series of interactions that occur with the sea and continents in different time and spatial frames. The costs of global warming arise from high temperatures produced by continuous damage to the environment, caused by sudden events in terms of climatic catastrophes [2]. The inherent problem is that there is considerable uncertainty associated to these two types of events [3]. Since global warming is a very complex problem and its signs are sometimes difficult to see, there is without a doubt a high level of uncertainty among population about the existence of global warming. 

Mexico is among the 70 countries with greater Greenhouse Gas Emissions (GGE) *per capita*, because it produces 0.96 tonnes of carbon dioxide every year that go into the atmosphere; 30.5% of the emissions are directly involved in activities of soil usage change and deforestation [4]. In this respect, animal and vegetal species, as yet endangered because of human activities, will be also endangered because of Climatic Change (CC).

Due to its geographic location and complex landscape Mexico is one of the countries with greater species diversity. Besides, many of the species in this country are endemic [5]. In the state of Chihuahua half of the ecosystems are arid and semi-arid zones. The potential of these zones has to be evaluated for a proper resource management, since these ecosystems are fragile and have a slow restoration [6]. 

The Chihuahua desert is, biologically speaking, one of the richest in the World. It covers an approximate area of 391,046 sq. mi., and approximately 30% of cacti species grow in this desert [5]. Cacti are important because of their endemism and their wide distribution in the American continent [7]. The cacti family, known collectively as cactus or cacti, is one of the most typical groups of succulents and most of them have spines [8]. Out of the total that exist in Mexico, about 35% have a risk status, and the Northwest region of the country houses the higher diversity and endemism, unfortunately this diversity is threatened due to man’s activities [6]. Cacti may be exploited in different ways, including as hedges and as cattle food, however their most common usage is as ornamental plants and as part of the human diet.

Tellez-Valdez and Davila-Aranda say that ecological niche modeling allows for the analysis of factors associated with different populations of a particular species with different degrees of impact [9]. The information that was analyzed by algorithms enables the projection in the geographical level of the potential area covered by the species. The results of the spatial studies may provide critical information about the diversity present on certain geographic areas and they can be used for different purposes, such as assessing of the current state of plant species conservation or prioritizing conservation areas [10].

There are general factors that affect species distribution: temperature, water availability and topography. More particularly they can be described as soil types, evapotranspiration, light quality or days with temperatures below the freezing point, among others [11]. It is important to know that niche modeling represents an approximation of the species’ ecological niche in the dimensions in the environmental crusts used [12]. 

There are modeling efforts where the MaxEnt program was applied, including those by Carroll who evaluated and modeled with the relations that exist between weather and vegetation variables, concluding that MaxEnt has a good performance in contrast with 15 alternative methods in a wide taxa variety in different regions. The variables measured were temperature and precipitation, provided for the three simulations of the future climate during two time periods (2011 to 2040 and 2061 to 2090) [13]. 

Colombo and Joly [14] worked on a zone in the Atlantic Forest. The data about species distribution were taken from Oliveira and Scudeller and Martins [15,16], who compared more than 100 lists of tree species, and 38 were selected for which there were not enough data about their current distribution. The selected variables were slope, diurnal temperature range, average annual precipitation and vapor pressure (annual averages 1960–1990). Regarding to future scenario projection, CC crusts were used for the next 50 years (IPCC 2001) and the same data for topographical issues, taking into account the little possibility of topographical changes in the next 50 years. It was concluded that all the MaxEnt projection models on the current area covered by the 38 species, present a high significance level (binomial test: two ratios, *p* < 0.05 for all species). Based on the above, the objective of the research reported herein was to evaluate the impact of the climate change on the geographical distribution of four cacti species found in the state of Chihuahua. 

## 2. Materials and Methods

### 2.1. Description of the Area of Study

The distribution areas for the selected species are found in the state of Chihuahua. The state is formed by three major regions: the Sierra, Plateau and Desert, which occur from west to east in the form of big stripes. This results in the greatly contrasting weather and geographical conditions that give the State its better known images, its great deserts, mountains, canyons and forests [5]. The Chihuahua desert is, biologically speaking, one of the richest in the World. This territory is mostly flat, though it has low sierras across, most of them north to south [17]. The desert weather in this region is very dry, yearly rainfall is less than 0.54 in. (350 mm); average temperatures go from 55.4 °F (13 °C) in January to 96.8 °F (36 °C) in June, reaching 122 °F (50 °C) in the hottest days of the year, with frosts in winter. Snow also appears in this region, though less frequently. 

### 2.2. Selected Species

Four cacti species with distribution on Chihuahua Desert were selected. These species were selected from collected previously registers, they are: *Coryphantha macromeris*, *Mammillaria lasiacantha*, *Echinocereus dasyacanthus* and *Ferocactus wislizenii* [8].

### 2.3. Modeling and Simulation

Taking into account the literature research of methodologies used already, the process to be followed for the species’ distribution modeling and simulation is described in the following paragraphs. It is necessary to collect bio-climatic crusts (these crusts were downloaded from WorldClim, with a resolution of 1 Km., for four periods of time 2000, 2020, 2050 and 2080, under three different scenarios A2, B1 y A1B [18]) and georefencered data. Georeferenced data are saved on spreadsheets such as MS Excel files in UTM format. The next step is to convert or save these data in formats that can be read with modeling niche programs. This task is made through the Geographic Information Systems (GIS) such as ArcMap with the option “Conversion Tools”, which allows the crusts to be saved in ASCII format so they can be read by MaxEnt [19]. Finally the MaxEnt program is run, taking geolocalities and climatic crusts data, it generates an area map of species’ potential location, and these files can be seen in GIS (ArcMap).

## 3. Results and Discussion

**Figure 1:**
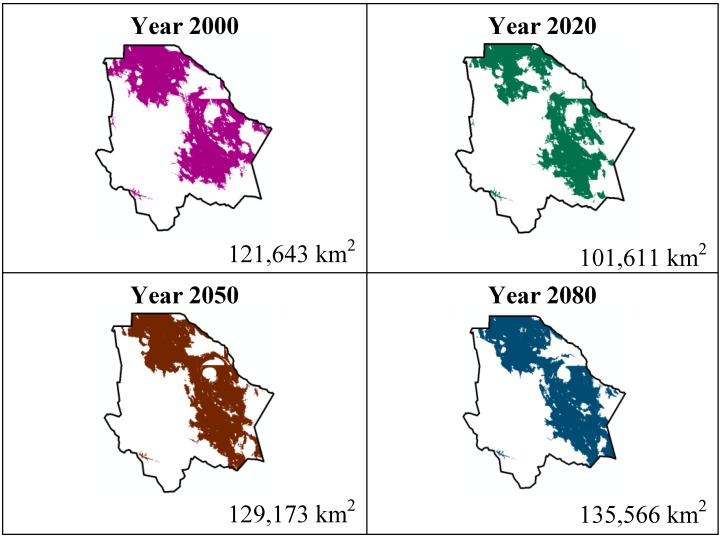
Potential distribution models (MaxEnt) for *Coryphantha macromeris* considering a liberal A2 temperature climatic scenario (current, 2020, 2050 and 2080).

**Figure 2:**
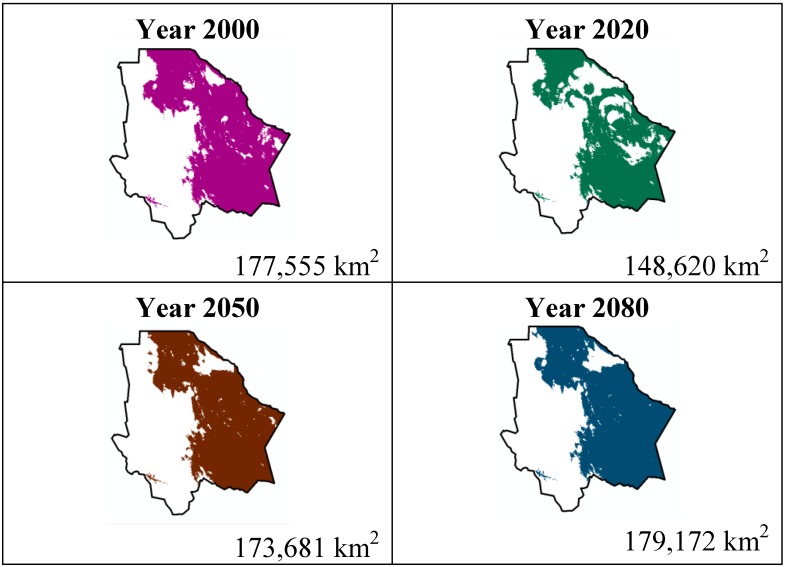
Potential distribution models (MaxEnt) for *Mamillaria lasiacantha* considering a liberal A2 temperature climatic scenario (current, 2020, 2050 and 2080).

**Figure 3:**
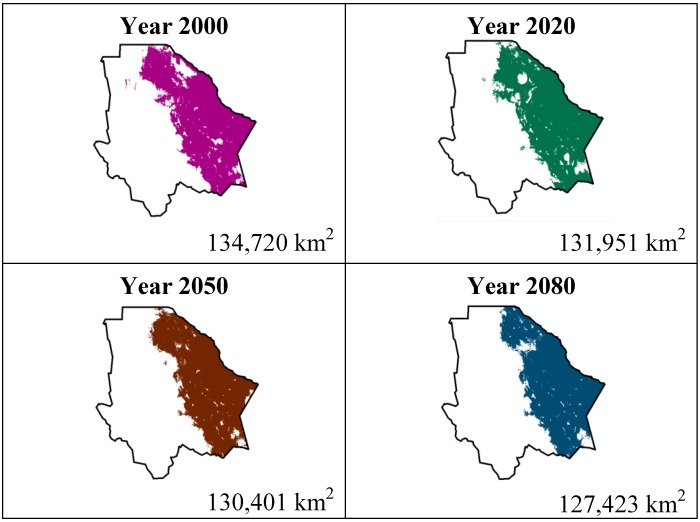
Potential distribution models (MaxEnt) for *Echinocereus dasyacanthus* considering a liberal A2 temperature climatic scenario (current, 2020, 2050 and 2080).

**Figure 4:**
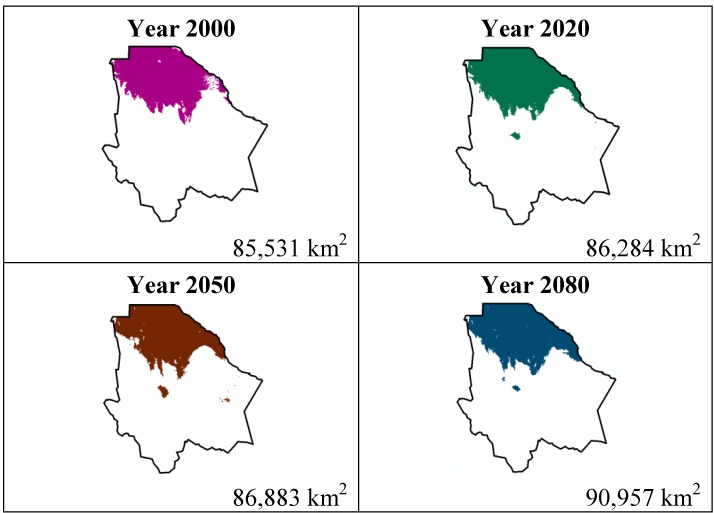
Potential distribution models (MaxEnt) for *Ferocactus wislizenii* considering a liberal A2 temperature climatic scenario (current, 2020, 2050 and 2080).

The data presented in Table 1 show the results from MaxEnt modeling. It is observed how the four cacti species will be located on scenario A2 (Figure 1, Figure 2, Figure 3 and Figure 4), with a decrease in relation to the potential area by the year 2020. This decrease can be taken as non-significant for the *Coryphantha macromeris* and *Mammillaria lasiacantha* species because in the years 2050 and 2080 they have a major increase in their potential area that in the case of *Coryphantha macromeris* is larger than it is currently.

**Table 1:** Current and future Potential Areas of the species in km^2^.

Scenario	A2	B1	A1B	A2	B1	A1B
	*Coryphantha macromeris*			*Mammillaria lasiacantha*		
2000	121,643	ND	ND	177,557	ND	ND
2020	101,611	126,127	107,183	148,620	175,365	180,743
2050	129,173	100,073	78,228	173,681	170,489	123,352
2080	135,566	112,298	138,631	179,172	174,472	157,051
	*Echinocereus dasyacanthus*			*Ferocactus wislizenii*		
2000	134,720	ND	ND	85,531	ND	ND
2020	131,951	147,397	128,736	86,285	79,520	67,551
2050	130,401	137,911	130,518	86,883	65,355	80,185
2080	127,423	136,181	33,789	90,957	75,627	78,144

Note: ND: Data non available for these scenarios.

Both species, *Echinocereus dasyacanthus* and *Ferocactus wislizenii*, present a decrease in their areas in the year 2050, unlike *Echinocereus dasyacanthus*, and the *Ferocactus wislizenii* species will increase its area in 2080, to larger than its current area. 

The study showed about the variable contribution percentage of the variables that temperature has a greater influence in species distribution. The variables were: 10 (Average temperature of the hotter quadrimester) more significant for *Coryphantha macromeris*; variable 1 (Average annual temperature) for *Mammillaria lasiacantha*; variable 8 (Average temperature of the rainiest quadrimester) for *Echinocereus dasyacanthus* and variable 7 (Annual tolerance range) for *Ferocactus wislizenii*. Taking into account that climate change causes an increase in the planet’s temperatures and being the temperature variables those with a greater influence on these cacti species, it is reasonable to say that the tolerance range for these species is wide since MaxEnt did not indicate a noticeable decrease about the species’ potential distribution area. 

**Figure 5:**
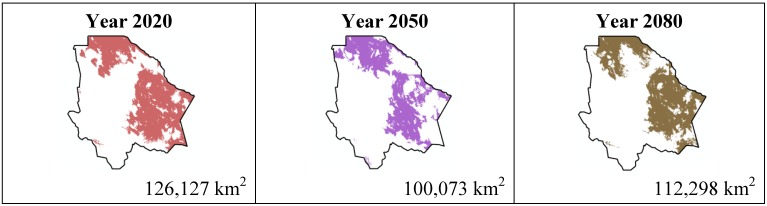
Potential distribution models (MaxEnt) for *Coryphantha macromeris* considering a liberal B2 temperature climatic scenario (current, 2020, 2050 and 2080).

**Figure 6:**
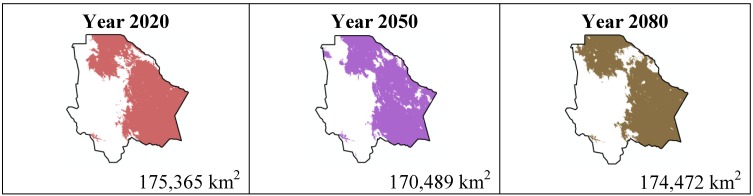
Potential distribution models (MaxEnt) for *Mamillaria lasiacantha* considering a liberal B1 temperature climatic scenario (current, 2020, 2050 and 2080).

**Figure 7:**
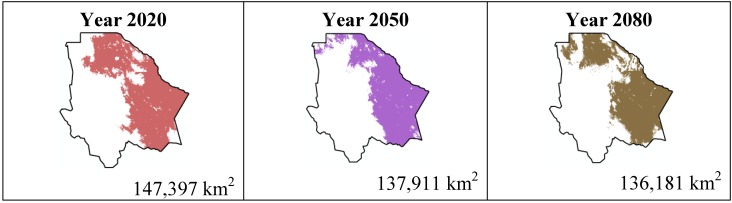
Potential distribution models (MaxEnt) for *Echinocereus dasyacanthus* considering a liberal B1 temperature climatic scenario (current, 2020, 2050 and 2080).

**Figure 8:**
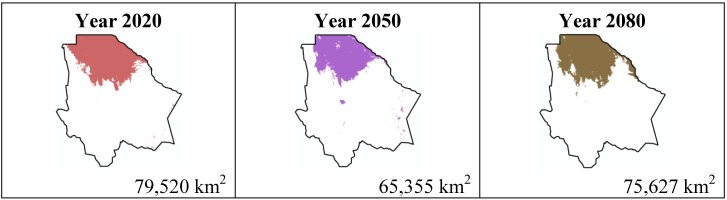
Potential distribution models (MaxEnt) for *Ferocactus wislizenii* considering a liberal B1 temperature climatic scenario (current, 2020, 2050 and 2080).

The results estimated for scenario B1 are similar to scenario A2 (Figure 5, Figure 6, Figure 7 and Figure 8); all species except *Echinocereus dasyacanthus* show a decrease from the year 2020 to the year 2050 and later in the year 2080 an increase in their distribution area. In the case of *Echinocereus dasyacanthus* the decrease in its potential distribution area is constant in the three time periods.

In spite of scenario B1 being about a reduction in the use of materials, a cleaner technology and a more efficient resource usage including global solutions to environmental sustainability, we can observe a difference about the species’ total distribution area for both scenarios. An important observation concerning the B1 scenario is that in the four cases the distribution area is smaller from year 2080 to 2020, where *Coryphantha macromeris* will decrease in 10.96%, *Mammillaria lasiacantha* in 0.5%, *Echinocereus dasyacanthus* in 7.6% and *Ferocactus wislizenii* in 4.89%.

The MaxEnt model shows an overview of the species spatial conditions, it is an approximation to reality that serves as starting point decision making in species conservation. Even though in the results we can see a resistance of cacti to CC, it is important to take into account other important factors such as soil usage or species trafficking that place them in risk.

**Figure 9:**
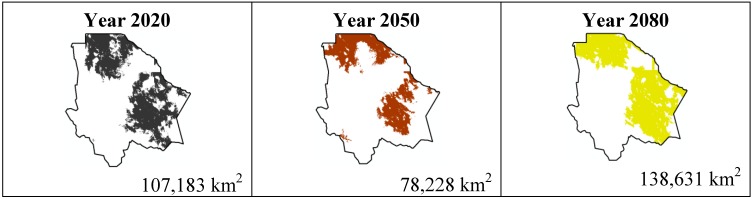
Potential distribution models (MaxEnt) for *Coryphantha macromeris* considering a liberal A1B temperature climatic scenario (current, 2020, 2050 and 2080).

**Figure 10:**
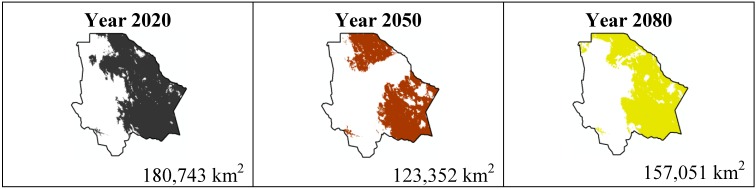
Potential distribution models (MaxEnt) for *Mamillaria lasiacantha* considering a liberal A1B temperature climatic scenario (current, 2020, 2050 and 2080).

**Figure 11:**
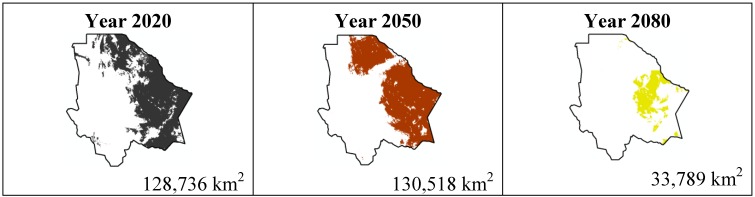
Potential distribution models (MaxEnt) for *Echinocereus dasyacanthus* considering a liberal A1B temperature climatic scenario (current, 2020, 2050 and 2080).

**Figure 12:**
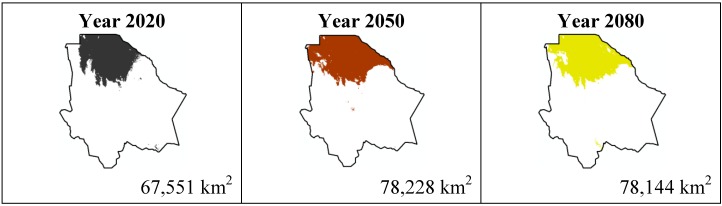
Potential distribution models (MaxEnt) for *Ferocactus wislizenii* considering a liberal A1B temperature climatic scenario (current, 2020, 2050 and 2080).

The most important results were projected by MaxEnt for the scenario A1B (Figure 9, Figure 10, Figure 11 and Figure 12). For *Coryphantha macromeris* it shows a decrease of 27% from the year 2020 to 2050, but the area will increase in 2080 to 86.14 sq. mi. which represents an increase of 22% from the projection for 2020. *Mammillaria lasiacantha* decreases from 2020 to 2050 and increases in 2080, being left with a distribution area 13% smaller than in 2020. *Echinocereus dasyacanthus* presented a decrease in the three time periods in scenario A2 as well as in scenario B1, this shows it as the only constant species in both scenarios, however for scenario A1B it will have a slight increase of 1.38% by 2050 and similar to the previous scenarios it will decrease, in this case 73% smaller than in 2020. Finally *Ferocactus wislizenii* increases in 15% from 2020 to 2050 and for 2080 it decreases 2.5%. 

The description given by IPCC for the A1B scenario is made as part of the A1 scenario where it is supposed that economic growth is fast, with an accelerated population growth that peaks in the twenty-first century and decreases afterwards; a world where there is a quick introduction of new and efficient technologies, especially in A1B which would have a balance of all energy sources.

**Figure 13:**
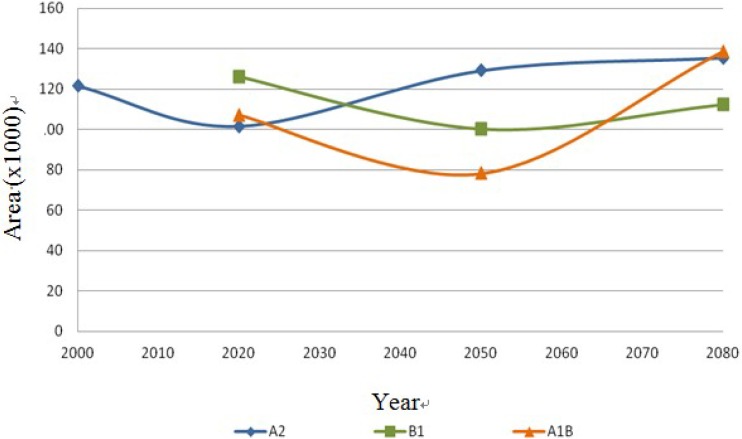
*Coryphantha macromeris* dispersal area on the different scenarios (A2, B1 and A1B) in km^2^.

**Figure 14:**
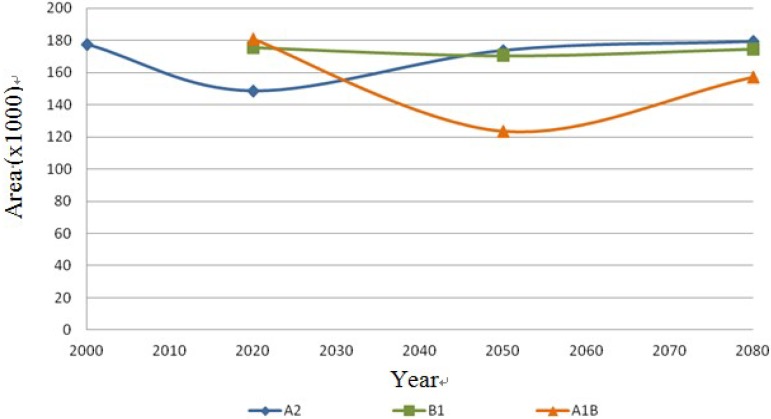
*Mammillaria lasiacantha* dispersal area on the different scenarios (A2, B1 and A1B) in km^2^.

**Figure 15:**
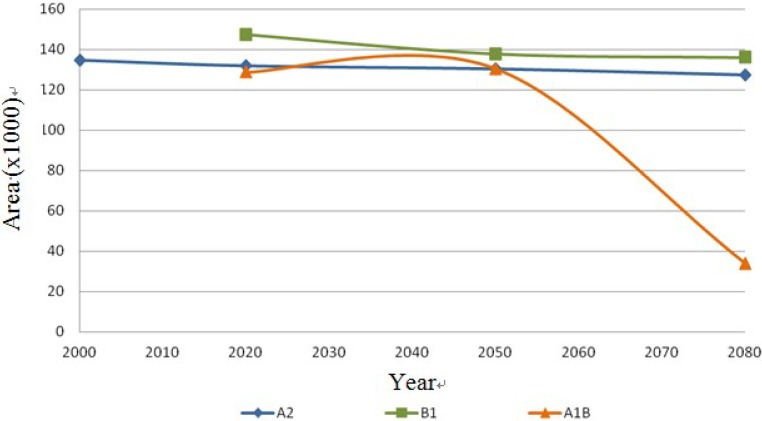
*Echinocereus dasyacanthus* dispersal area on the different scenarios (A2, B1 and A1B) in km^2^.

**Figure 16:**
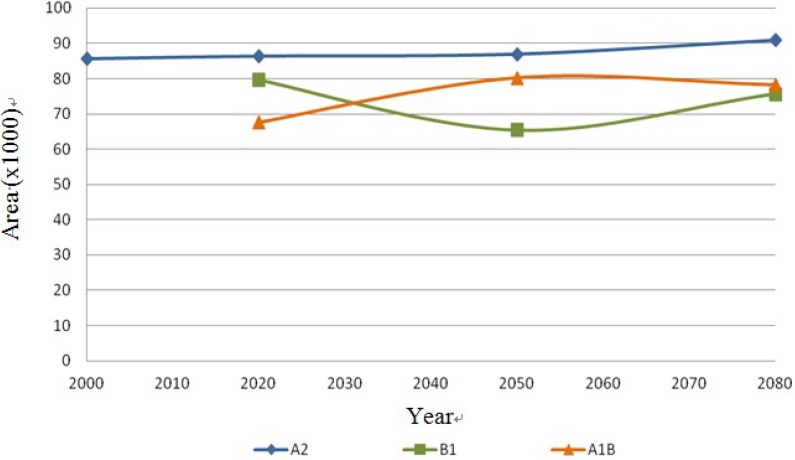
*Ferocactus wislizenii* dispersal area on the different scenarios (A2, B1 and A1B) in km^2^.

The following Figure 13, Figure 14, Figure 15 and Figure 16 show the behavior of the four species on the IPCC different scenarios and time periods.

## 4. Conclusions

The results show stability regarding the present and future distribution of cacti species under scenarios A2 and B1, and the most significant changes were for scenario A1B. In the first scenario A2, the species *Coryphantha macromeris* decreases 16.46% in the first period of time from 2000 to 2020, but for the following periods it increases 25.04% from 2020 to 2080. In scenario B1 it decreases 20.65% from the year 2020 to 2050, ending in 2080 with 10.88% more. Finally this species in the scenario A1B presents a decrease of 27.01% in its distribution area from 2020 to 2050 and it has an increase of 43.57% by 2080. The species *Mammillaria lasiacantha* presents a decrease of 16.29% in the period 2000–2020 for the case of the first scenario A2; however both in the period of 2020–2050 and 2050–2080 it presents an area increase of 14.42% and 3.06% respectively. In the case of scenario B1 the species has a decrease for the first period but for the following years it increases, projecting a loss of only 0.5% from 2020 to 2080. In the third scenario the results of the distribution area present like to the previous one a decrease in the first period from 2020 to 2050 of 31.75% and it repeats the same pattern increasing 21.45% in the second period from 2050 to 2080. The third species *Echinocereus dasyacanthus* presents in the first scenario A1, a decrease of 5.41% from 2000 to 2080. The behavior of this species in the scenario B1 is the same as in the previous scenario with a decrease from 2020 to 2080, being in this case of 7.60%. In the following scenario A1B this species shows an increase in its area of 1.36% in the first period 2020–2050, but the decrease in the second period 2050‑2080 is significant, with 74.11% of its potential distribution area. Finally, the species *Ferocactus wislizenii* presents an increase of 5.96% from 2000 to 2080 in the first scenario A2. The scenario B1 presents a decrease of 17.81% in the first period 2020–2050 and it increases 13.58% in the final period 2050–2080. The scenario A2B for this species starts with an increase of 17.81% in the first period 2020–2050 and it decreases 2.54% in the final period 2050–2080.

Although cacti distribution was not affected significantly by climate change, we should continue research to know the state of other species in Mexico. It is also important to take into account other factors such as changes in soil usage that affect the species distribution directly, decreasing their distribution.

Since damage can be done silently, slowly and many times continuously; at first sight the alteration is not evident. MaxEnt allows for the diagnosis of the present and/or future to envision possible solutions to preserve these species. It is necessary to analyze the effects that climate change may have on the different species distribution areas. 
